# Coffee and Endothelial Function: A Coffee Paradox?

**DOI:** 10.3390/nu11092104

**Published:** 2019-09-04

**Authors:** Yukihito Higashi

**Affiliations:** 1 Department of Cardiovascular Regeneration and Medicine, Research Institute for Radiation Biology and Medicine, Hiroshima University, 1-2-3 Kasumi, Minami-ku, Hiroshima 734-8551, Japan; yhigashi@hiroshima-u.ac.jp; Tel./Fax: +81-82-257-5831; 2 Division of Regeneration and Medicine, Medical Center for Translational and Clinical Research, Hiroshima University Hospital, Hiroshima 734-8551, Japan

**Keywords:** coffee, caffeine, endothelial function, nitric oxide

## Abstract

Coffee is a popular beverage throughout the world. Coffee contains various chemical compounds (e.g., caffeine, chlorogenic acids, hydroxyhydroquinone, kahweol, cafestol, and complex chemical mixtures). Caffeine is also the most widely consumed pharmacological substance in the world and is included in various beverages (e.g., coffee, tea, soft drinks, and energy drinks), products containing chocolate, and drugs. The effects of coffee and caffeine on cardiovascular diseases remain controversial. It is well known that there are J-curve-type or U-curve-type associations of coffee consumption with cardiovascular events including myocardial infarction and stroke. However, there is little information on the direct and indirect effects of coffee consumption on endothelial function in humans. It is likely that the coffee paradox or caffeine paradox exists the association of coffee intake with cardiovascular diseases, cardiovascular outcomes, and endothelial function. This review focusses on the effects of coffee and caffeine on endothelial function from molecular mechanisms to clinical perspectives.

## 1. Introduction

Coffee is a popular beverage that contains various chemical compounds including caffeine, chlorogenic acids (CGA), hydroxyhydroquinone (HHQ), kahweol, cafestol, and complex chemical mixtures [1,2], and it is the most widely consumed beverage used in the world. Epidemiologic studies have shown inverse associations of regular consumption of coffee with metabolic disorders including obesity, metabolic syndrome, and type 2 diabetes mellitus, as well as with cardiovascular diseases (CVD) [3,4,5,6,7,8]. However, there are conflicting results concerning the associations of coffee consumption with risks of CVD, including coronary artery disease, hypertension, heart failure, and arrhythmia [9,10,11,12,13,14,15]. Interestingly, it has been shown that there are J-curve-type and U-shaped-type associations of coffee intake with myocardial infarction and stroke [16,17]. Caffeine is also the most widely consumed pharmacological substance in the world. It is included in various beverages (e.g., coffee, tea, and soft drinks), chocolate, and drugs. Several lines of evidence have shown that caffeine is associated with an increased incidence of cardiovascular (CV) outcomes [9,10,11]. On the other hand, a recent epidemiological study has shown beneficial effects of caffeine on CVD [3], while other studies have shown that there is no relationship between an increasing level of caffeine consumption and risks of CV morbidity and mortality [12,13]. Thus, the association of caffeine with the development of CVD is controversial. There have been many studies on the effect of caffeine on blood pressure in patients with hypertension [18,19,20,21,22,23]. Acute ingestion of caffeine has been shown to elevate blood pressure via elevation of peripheral vascular resistance [18,19,20,21], but meta-analysis studies have shown that chronic intake of caffeine has a smaller effect on blood pressure [22,23]. Thus, the effect of caffeine on blood pressure is also controversial.

It is well known that endothelial dysfunction is the initial step in the pathogenesis and development of atherosclerosis, which results in hypertension and other CVD [24,25]. Several investigators have demonstrated the effects of various interventions on endothelial function in the brachial or coronary arteries [26,27,28,29,30]. In most of the studies in which the relationship between coffee consumption and endothelial function was evaluated, coffee augmented the endothelial function in healthy subjects and improved endothelial function in patients with CVD [31,32,33,34,35,36,37,38,39,40,41,42,43,44]. However, some studies showed that coffee consumption impaired endothelial function [45,46,47], while other studies showed that there is no association of coffee consumption with endothelial function [48,49,50]. Thus, the relationship between coffee consumption and endothelial function is also controversial. In addition, unfortunately, there is a little information on the direct effects of caffeine on endothelial function.

This review focuses on the effects of coffee and caffeine on endothelial function from molecular mechanisms to clinical perspectives.

## 2. Role of Endothelial Function in Atherosclerosis

Endothelial cells secrete a number of vasoactive chemicals including vasodilators—such as nitric oxide (NO), prostacyclin, and endothelium-derived hyperpolarizing factor (EDHF)—and vasoconstrictors—such as angiotensin II, thromboxane A_2_, and endothelin-1 [51,52,53]. It is well known that a healthy endothelium maintains vascular tone and structure by regulating the balances of anti-oxidation and pro-oxidation, anti-inflammation and pro-inflammation, anti-thrombosis and pro-thrombosis, growth inhibition and growth promotion, and vasodilation and vasoconstriction [51,52,53]. Impairment of endothelial function is the initial step in the pathogenesis of atherosclerosis and plays a critical role in the maintenance and development of atherosclerosis [24,25]. If a healthy endothelium is impaired and then changes to a state of endothelial dysfunction, a vicious circle will arise between endothelial dysfunction and atherosclerosis. Several lines of evidence have shown that endothelial function is impaired in the brachial, forearm, coronary, leg, and renal arteries in subjects with coronary risk factors and in patients with CVD [26,27,28,29,30,54,55,56,57,58,59,60,61,62,63,64]. Some interventions, such as pharmacological therapy, lifestyle modifications, and supplementation therapy, but not all interventions, improve or augment endothelial function, suggesting that endothelial dysfunction is reversible. In addition, previous studies have shown that assessment of endothelial function and assessment of vascular smooth muscle function are independent predictors of CV events [65,66,67,68,69,70,71,72]. It has been proposed that the mechanisms of endothelial dysfunction and vascular smooth muscle dysfunction involve an inactivation of NO by reactive oxygen species (ROS), inflammation, increases in vasoconstrictors, an increase in the endogenous endothelial NO synthase (eNOS) inhibitor asymmetrical dimethylarginine, and an abnormality of shear stress [25,73,74].

## 3. Endothelial Function Test

Since the first assessment of endothelial function in humans in 1986, endothelial function has been assessed using several methods [75]. In a clinical setting, assessment of endothelial function using an established method is important. Methods that have been used for the assessment of endothelial function in humans include the measurement of endothelium-dependent vasodilation induced by intra-arterial infusion of a vasoactive agent by using coronary angiography, a Doppler flow guide wire [65,75], or plethysmography [25,66]; measurement of flow-mediated vasodilation (FMD) using ultrasonography [69,70,71]; measurement of reactive hyperemia index (RHI) using RH-peripheral arterial tonometry [72]; and measurement of enclosed zone FMD (ezFMD) using an oscillometric method [76]. At present, measurement of vascular responses to intra-arterial infusion of NO agonists and NO antagonists in the coronary and brachial arteries is the gold standard for the assessment of endothelial function. However, this technique is invasive and is burdensome for the subjects. Recently, noninvasive methods, namely measurements of FMD and RHI, have been widely used worldwide. Measurements of FMD and RHI are noninvasive, simple, and reproducible. Measurement of vascular response to exogenous NO in the coronary and brachial arteries is useful not only for assessment of endothelium-independent vasodilation (vascular smooth muscle function), but is also a control test for endothelial function. Measurements of vascular function, endothelium-dependent vasodilation, and endothelium-independent vasodilation are useful for the assessment of the grade of atherosclerosis and are predictors of CV events [65,66,67,68,69,70,71,72]. It is important to know the advantages and disadvantages of each method for assessing endothelial function and vascular smooth muscle function.

## 4. Effects of Coffee and Caffeine on Endothelial Function

The effects of coffee and caffeine on endothelial function in humans are summarized in Table 1. Although tea has many anti-oxidant properties, the effects of black tea and green tea on endothelial function are also shown in Table 1 since tea contains caffeine. As shown in Table 1, the acute and chronic effects of coffee and caffeine on endothelial function were examined in healthy subjects, subjects with CV risk factors, and patients with CVD using several endothelial function tests [31,32,33,34,35,36,37,38,39,40,41,42,43,44,45,46,47,48,49,50,77,78,79,80,81,82,83,84,85,86].

### 4.1. Coffee

The effects of coffee ingestion on endothelial function are quite controversial. Ingestion of coffee was shown to have beneficial effects on endothelial function in 14 studies [31,32,33,34,35,36,37,38,39,40,41,42,43,44], harmful effects on endothelial function in three studies [45,46,47], and no effects on endothelial function in three studies [48,49,50].

In the 14 studies showing augmentation or improvement of endothelial function after coffee intake, the effects of coffee containing CGA from low to high doses (89 mg to 412 mg or no description of exact doses of CGA and caffeine) on FMD, RHI, acetylcholine (ACh)-induced vasodilation, and chemical biomarkers were evaluated [31,32,33,34,35,36,37,38,39,40,41,42,43,44]. In 9 of the 14 studies, endothelial function was assessed using FMD of the brachial artery after ingestion of coffee containing CGA at doses from 89 mg to 412 mg and with caffeine from 54.5 mg to 126 mg in healthy young subjects (*n* = 13 to 19, follow-up of 1 h to 2 weeks) and healthy elderly subjects (*n* = 142, cross-sectional study), subjects with CV risk factors (*n* = 21, follow-up of 8 weeks), and patients with mild hypertension (*n* = 37, follow-up of 1 week) [31,32,33,34,35,36,37,38,39]. Coffee beans are roasted, resulting in a loss of CGA, an antioxidative substance, and generation of HHQ, an oxidative substance [87]. Kajikawa et al. evaluated the effects of ingestion of coffee with a high content of CGA and a low content of HHQ for 7 days on vascular function after meal loading in 37 patients with borderline or stage 1 hypertension using a single-blind, randomized, placebo-controlled, crossover-within-subject clinical trial [44]. Coffee with a high content of CGA and a low content of HHQ, but not with a high content of CGA and a high content of HHQ or placebo coffee, significantly improved postprandial FMD. In 3 of the 14 studies, endothelial function was assessed using the response to reactive hyperemia of finger circulation after ingestion of coffee containing 89 mg of CGA and 54.5 mg of caffeine (no description of exact doses of CGA and caffeine in two reports) by healthy subjects (*n* = 15, follow-up of 1.5 h; *n* = 20, follow-up of 4 months; and *n* = 27, follow-up of 75 min) [32,36,39]. Tesselaar et al. evaluated forearm skin vasodilatory responses to an endothelium-dependent vasodilator, Ach, and to an endothelium-independent vasodilator, sodium nitroprusside (SNP), in 16 healthy subjects before and after acute oral administration of caffeinated coffee containing 78 mg of caffeine or decaffeinated coffee in a double-blind, randomized, placebo-controlled, crossover study [42]. Caffeinated coffee, but not decaffeinated coffee, significantly augmented the forearm skin vasodilatory responses to ACh, while sodium nitroprusside (SNP)-stimulated vasodilation was not altered by the administration of caffeinated coffee or decaffeinated coffee administration. Lopez-Garcia et al. carried out a cross-sectional study to determine the regular effects of caffeinated or decaffeinated coffee consumption (<1 cup/month, 1 cup/month to 4 cups/week, 5–7 cups/week, and ≥2 cups/day) on markers of endothelial dysfunction in 730 healthy women and 663 type 2 diabetic women from the Nurses’ Health Study 1 cohort [31]. They found that caffeinated coffee consumption, but not decaffeinated coffee consumption, was inversely correlated with a marker of endothelial dysfunction E-selectin in type 2 diabetic women, while there were no significant relationships between caffeinated coffee or decaffeinated coffee consumption and markers of endothelial function in healthy women.

Three studies showed impairment of endothelial function after coffee ingestion. Two studies showed harmful effects of coffee ingestion on endothelial function assessed using a physiological function test, namely FMD measurement [45,46,47]. Papamichael et al. evaluated FMD in 17 healthy subjects before and after acute oral administration of coffee containing 80 mg of caffeine [45]. Buscemil et al. evaluated FMD in 20 healthy subjects before and after 5–7 days of oral administration of 25 mL of Italian espresso coffee [46]. Bruce et al. showed that acute oral administration of coffee containing 100 mg of caffeine, but not acute oral administration of decaffeinated coffee, decreased exhaled NO levels in 12 healthy subjects in a single-blind, randomized, placebo, and cross-over study [47].

Three studies showed no effects of coffee ingestion on endothelial function [48,49,50]. Molnar et al. evaluated the RHI of finger tips in 19 healthy subjects before and after acute oral administration of coffee containing 200 mg of caffeine [48]. Agudelo-Ochoa et al. evaluated FMD in 74 healthy subjects before and after 8 weeks of oral administration of coffee containing 193 mg of caffeine and 420 mg to 780 mg of CGA [49]. Ward et al. evaluated FMD in 16 healthy subjects before and after acute oral administration of coffee containing 450 mg to 900 mg of CGA [50]. The latter two studies showed that the ingestion of coffee, even coffee containing a high dose of CGA, had no effect on endothelial function.

### 4.2. Caffeine

It is hard to evaluate the direct effects of caffeine on endothelial function using caffeinated coffee since coffee has a number of anti-oxidant and pro-oxidant properties and anti-inflammation and pro-inflammation properties. There have been only three studies in which the direct effect of caffeine intake on endothelial function in humans was evaluated [77,78,79]. In the first study, Umemura et al. evaluated forearm blood flow (FBF) responses to ACh and SNP by using strain-gauge plethysmography in healthy young men before and after acute oral administration of 300 mg of caffeine (*n* = 10) or a placebo (*n* = 10) in a double-blind, randomized, placebo, and active drug study [77]. Caffeine significantly increased both systolic and diastolic blood pressures but did not alter heart rate and baseline FBF. Caffeine augmented the ACh-induced vasodilation, while SNP-induced vasodilation was not altered by caffeine administration. Intra-arterial infusion of an NOS inhibitor *N*^G^-monomethyl-L-arginine (L-NMMA) abolished the caffeine-induced augmentation of vasodilatory response to ACh. Both the ACh-induced vasodilation and SNP-induced vasodilation were similar in the placebo group before and after the follow-up period. In the second study, Shechter et al. evaluated FMD and NID before and after acute oral administration of 200 mg of caffeine in patients with coronary artery disease (CAD; *n* = 40) and subjects without CAD (*n* = 40) in a randomized, double-blind, placebo-controlled, cross-over study [78]. They showed that caffeine ingestion significantly improved FMD but not NID in both patients with CAD and subjects without CAD. In the third study, Alexopoulos et al. evaluated FMD before and after acute oral administration of green tea containing 125 mg of caffeine or only 125 mg of caffeine in 14 healthy subjects [79]. Green tea significantly augmented FMD, while an equivalent dose of caffeine did not alter FMD. This was not a double-blind, randomized, placebo study. Bruce et al. showed that exhaled NO levels decreased during a period of 3 h after acute oral administration of 200 mg of caffeine in 12 healthy subjects in a single-blind, randomized, placebo, and cross-over study [45].

### 4.3. Tea

All of the studies using black tea or green tea showed significant augmentation of FMD in healthy subjects and significant improvement of FMD in subjects with coronary risk factors and patients with CAD, while the effects of tea on NID were controversial (Table 1) [80,81,82,83,84,85,86].

## 5. Mechanisms of the Effects of Coffee and Caffeine on Endothelial Function

**Figure 1:**
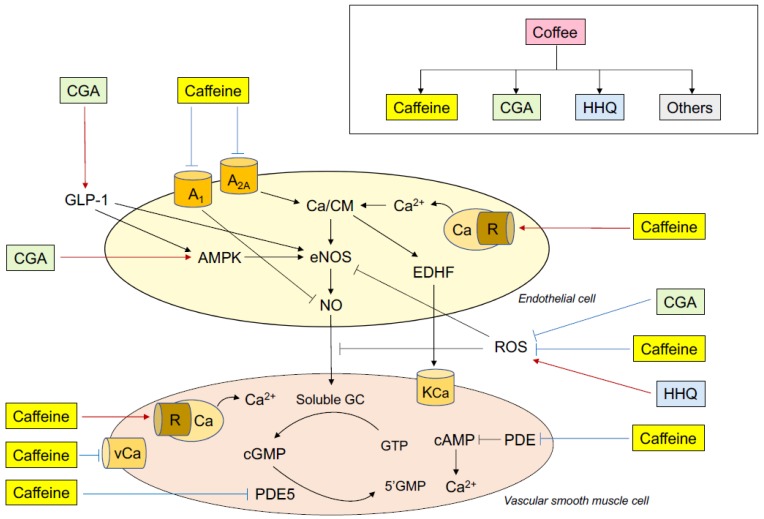
Putative mechanisms of the effects of coffee and caffeine on endothelial function. CGA indicates chlorogenic acids; HHQ, hydroxyhydroquinone; A_1_, adenosine receptor A_1_; A_2A_, adenosine receptor A_2A_; Ca, calcium; R, ryanodine receptor; GLP-1, glucagon-like peptide-1; AMPK, adenosine monophosphate-activated protein kinase; eNOS, endothelial nitric oxide synthase; EDHF, endothelium-derived hyperpolarizing factor; NO, nitric oxide; ROS, reactive oxygen species; KCa, calcium-activated potassium channel; vCa, voltage-dependent calcium channel; GC, guanylate cyclase; cGMP, cyclic guanosine monophosphate; GTP, guanosine triphosphate; PDE5, phosphodiesterase 5.

Some possible mechanisms by which ingestion of coffee and ingestion of caffeine influence endothelial function in humans are postulated (Figure 1). Both coffee ingestion and caffeine ingestion augment or improve endothelial function and diminish or impair endothelial function via the balances between vasodilation and vasoconstriction, anti-thrombosis and pro-thrombosis, anti-inflammation and pro-inflammation, and anti-oxidation and pro-oxidation.

### 5.1. Coffee

Coffee contains vascular regulatory compounds including anti-oxidant substance CGA and pro-oxidant substance HHQ, as well as caffeine [1,2]. Therefore, it is thought that these compounds are complexly intertwined after coffee ingestion to regulate endothelial function. The role of caffeine in endothelial function is more complex since caffeine works as a NO stimulator, NO inhibitor, and inhibitor of NO second messenger cyclic guanosine monophosphate (cGMP) degradation (see Section 5.2). This section focuses on the roles of CGA and HHQ in endothelial function.

It is well known that oxidative stress induced by an imbalance of NO and reactive oxygen species (ROS) plays a critical role in the pathophysiology, maintenance, and development of endothelial dysfunction through a decrease in NO bioavailability [25,73,74]. Fourteen of the 20 studies on the effects of coffee ingestion on endothelial function showed that acute and chronic coffee ingestion augments or improves endothelial function [31,32,33,34,35,36,37,38,39,40,41,42,43,44]. However, three studies showed that coffee does not alter endothelial function and three studies showed that coffee diminishes or impairs endothelial function [48,49,50]. Although the precise reasons for the discrepant results are unclear, it is possible that HHQ cancels the beneficial effects of CGA on endothelial function or exhibits performance-exceeding oxidative stress via an increase in the production of ROS. Indeed, Kajikawa et al. showed that ingestion of coffee with a high content of CGA and low content of HHQ, but not an intake of coffee with a high content of CGA and high content of HHQ, improved postprandial endothelial dysfunction assessed using FMD through a decrease in oxidative stress [44]. In spontaneously hypertensive rats, Suzuki et al. showed that HHQ inhibited the CGA-induced improvement in endothelial function via an increase in the production of ROS in a dose-dependent manner [88]. CGA act as direct scavengers of ROS due to their antioxidant property. Jiang et al. showed that CGA improved oxidative-stress-induced endothelial dysfunction in mouse aorta rings and protected endothelial cells in mice through the production of NO and the induction of heme oxygenase-1 (HO-1) [89]. HO is a rate-limiting enzyme that degrades pro-oxidative heme to carbon monoxide, ferrous iron, and biliverdin, which is rapidly converted to bilirubin by biliverdin reductase [90]. Carbon monoxide and unconjugated bilirubin have been demonstrated to have anti-inflammatory and anti-oxidative activities, although they have cytoprotective effects under various stressful conditions, including inflammation and oxidative stress [91,92]. HO-1 expression is known to be enhanced by the nuclear factor erythroid 2-related factor 2 (Nrf2)/antioxidant response element pathway [93,94]. CGA modulates Nrf2 nuclear translocation and antioxidant-response-element-related gene expression [95]. Under the condition of oxidative stress, CGA may contribute to the protection of endothelial dysfunction through activation of the HO-1/NO/eNOS pathway. These findings suggest that CGA directly scavenge ROS and activates eNOS, leading to augmentation or improvement of endothelial function. It has been shown that HHQ plays a critical role in the production of ROS, including hydrogen peroxide and superoxide anions, via an electron spin resonance method in a neutral solution of HHQ [96]. In addition, it has been shown that HHQ in coffee is a source of ROS in humans [97]. Several studies have shown that ROS inhibit not only NO production from the endothelium but also intravascular signaling processes in vascular smooth muscle cells by inhibiting the activity of soluble guanylyl cyclase and cGMP-dependent kinase, leading to vascular smooth muscle dysfunction, and consequently, impaired vasodilatory responses to nitroglycerine and SNP [98,99]. It is likely that a ROS-related decrease in NO bioavailability via an imbalance between CGA and HHQ regulates endothelial function after coffee ingestion (Figure 1). These findings suggest that careful attention should be given to the ratio of CGA to HHQ in coffee for assessing vascular function and cardiovascular events.

The effects of coffee consumption on inflammation are also controversial. Lopez-Garcia et al. showed that consumption of caffeinated coffee, but not consumption of decaffeinated coffee, is inversely correlated with C-reactive protein (CRP), an inflammation marker, in type 2 diabetic women, while there was no significant association of caffeinated coffee or decaffeinated coffee consumption with CRP in healthy women [31]. They found that a marker of endothelial function decreased in relation to the increase in coffee intake from <1 cup/month to ≥2 cups/day. Their findings suggest that coffee consumption of up to 2 cups/day has no harmful effects on endothelial function and inflammation, even if there is a possibility of regular consumption of coffee having beneficial effects on endothelial function and inflammation. In contrast, Zampelas et al. showed by conducting a cross-sectional study that regular consumption of >200 mL/day of coffee significantly increased circulating inflammation markers, including interleukin 6, CRP, amyloid-A, and tumor necrosis factor alpha, in 1514 men and 1528 women from the ATTICA study registry, suggesting that relatively moderate to high coffee consumption may cause inflammation [100]. It has been shown that CGA has a strong anti-inflammatory property [101,102]. Inflammation also plays a critical role in the impairment of endothelial function [103,104]. It is unlikely that the anti-inflammation induced by CGA contributes to the improvement of endothelial function. Unfortunately, there are no data on the direct effects of HHQ and inflammation from in vitro, in vivo, and clinical studies.

A meta-analysis showed antihypertensive effects of CGA [105]. HHQ diminished the CGA-induced decrease in blood pressure in spontaneous hypertensive rats [88]. Although there has been no study showing the long-term effect of ingestion of coffee with a high content of CGA and low content of HHQ on endothelial function, long-term intake of coffee with a high content of CGA and low content of HHQ was shown to be effective for decreasing blood pressure in patients with mild hypertension [33,106]. However, Kajikawa et al. showed that acute intake of coffee with a high content of CGA and low content of HHQ had no effect on blood pressure in patients with mild hypertension [44]. Acute intake of caffeine is associated with the acute elevation of blood pressure [88,107,108]. On the other hand, chronic caffeine consumption had no effect on blood pressure [107,108]. Blood pressure is a predictor of endothelial function. Therefore, in acute ingestion and chronic consumption of coffee, interaction among CGA, HHQ, and caffeine may alter blood pressure, leading to changes in endothelial function.

Adenosine monophosphate-activated protein kinase (AMPK) is ubiquitously present in various tissues and participates in the regulation of cellular energy homeostasis [109,110]. AMKP is also a key enzyme for the regulation of vascular homeostasis [111]. AMKP in endothelial cells is stimulated by metabolic stress; hypoxia stress; physical exercise; hormones including adiponectin, leptin, and estrogen; vasoactive agents including bradykinin, histamine, and thrombin; and pharmacological agents including statins and metformin [109,110,112]. Previous studies showed that GGA directly enhance the phosphorylation of AMPK in human umbilical vein endothelial cells (HUVEC) [113], as well as skeletal muscle cells of mice [114]. Pretreatment with CGA restored oxLDL-induced oxidative stress and mitochondrial dysfunction in HUVEC through activation of the AMPK/peroxisome proliferator-activated receptor γ coactivator-1 pathway and enhancement of Sirt-1 activity [113]. AMPK phosphorylates the serine residue 1177 of eNOS, which leads to eNOS activation, resulting in the production of NO from endothelial cells [115]. Therefore, the AKMP/eNOS pathway is one of the regulatory systems of endothelial function. These findings suggest that CGA augment or improve endothelial function through an increase in NO bioavailability via the dual manner of decreasing NO inactivation and increasing NO production.

Glucagon-like peptide-1 (GLP-1) is a gut hormone secreted from the intestine in response to meal ingestion and it improves blood glucose utilization and insulin resistance via the stimulation of insulin secretion and inhibition of glucagon secretion [116]. In addition, GLP-1 may have the ability to protect and/or improve vascular function. Decaffeinated coffee, but not caffeinated coffee, decreased circulating glucose-dependent insulinotropic peptide levels and increased circulating GLP-1 levels compared with those in healthy subjects as controls, suggesting that CGA, the most prevalent polyphenols in coffee, but not caffeine, contribute to the increase in GLP-1 secretion [117]. Fujii et al. showed that coffee polyphenols increased GLP-1 release from human enteroendocrine NCl-H716 cells in a dose-dependent manner through the cAMP pathway and increased circulating levels of GLP-1 in mice [118]. It has been shown that GLP-1 increases eNOS mRNA levels and increases eNOS activity in HUVEC through activation of the GLP-1(9–36)/GLP-1 receptor pathway [119]. GLP-1 or GLP-1(9–36) increased coronary blood flow and induced vasodilation of mesenteric arteries in mice through activation of the NO/eNOS/cGMP pathway [120]. The GLP-1 analog exenatide significantly improved endothelial function in coronary circulation of patients with type 2 diabetes and increased NO production through activation of the phosphatidylinositol-3 kinase (PI3K)/Akt/AMPK/eNOS pathway via a GLP-1 receptor-dependent mechanism in a dose-dependent manner in HUVEC [121]. In type 2 diabetes patients with coronary artery disease, intravenous infusion of GLP-1 significantly increased FMD but not insulin resistance [122]. These findings suggest that the CGA-induced increase in GLP secretion contributes to the augmentation or improvement of endothelial function after coffee ingestion.

The acute effects of only CGA and CGA with different HHQ contents on endothelial function have been evaluated in most studies. In future studies, long-term interventions should be performed to determine the long-term effects of coffee with a high content of CGA and low content of HHQ on endothelial function.

Although the ingredients of coffee differ depending on the kind of coffee beans, about 10 ingredients are generally included in coffee beans [123,124,125]. Green coffee beans contain 50.0–60.0% carbohydrates, 12.0–18.0% lipids, 11.0–13.0% proteins, 6.0–8.0% oligosaccharides, 6.0–8.0% polyphenols (CGA), 3.0–4.0% mineral, 1.0–2.0% fatty acids, 1.0–2.0% free amino acids, 1.0–2.0% trigonelline, and 0.5–1.5% caffeine [123,124,125]. After roasting coffee beans, the ingredients of coffee beans and the amount of coffee beans are changed to 25.0–40.0% carbohydrates, 15.0–20.0% lipids, 13.0–15.0% proteins, 0–4.0% oligosaccharides, 1.0–2.0% polyphenols (CGA), 3.0–5.0% minerals, 1.0–2.0% fatty acid, 0% free amino acids, 0.5–1.0% trigonelline, 0.5–1.0% caffeine, and 15.0–25.0% melanoidins [123,124,125]. A cup of medium-roasted coffee (100 mL) contains 500–1500 mg melanoidins, 200–800 mg soluble fiber, 250–700 mg minerals, 35–500 mg polyphenols (CGA), 50–400 mg caffeine, 100–200 mg proteins, 40–50 mg trigonelline, 1–10 mg niacin, and 1 mg lipids [123,126,127]. Coffee contains a large number of compounds that might influence endothelial function. The possibility that compounds other than CGA, HHQ, and caffeine have a greater impact on endothelial function cannot be excluded.

### 5.2. Caffeine

Coffee ingestion has been used in many studies to investigate the effects of caffeine. Coffee is the most widely consumed beverage containing caffeine. However, coffee contains not only caffeine but also various substances, such as carbohydrates, proteins, lipids, glycosides, and minerals, that may affect systemic and forearm hemodynamics [128]. Therefore, it is clinically important to use only caffeine to exclude effects of other factors. Three studies in which the direct effects of caffeine on endothelial function were evaluated showed that acute caffeine ingestion augmented and improved endothelium-dependent vasodilation but not endothelium-independent vasodilation in healthy subjects and patients with CAD, suggesting that caffeine selectively augments and improves endothelial function but not vascular smooth muscle function [77,78,79]. Umemura et al. showed that intra-arterial infusion of eNOS inhibitor L-NMMA completely abolished caffeine-induced augmentation of the FBF response to ACh [77]. These findings suggest that acute administration of caffeine augments endothelial function though an increase in NO production. On the other hand, Bruce et al. showed that acute oral administration of both coffee containing caffeine and only caffeine decreased exhaled NO, suggesting that caffeine decreases NO production from endothelial cells [45]. These discrepant results may be due to the different actions of caffeine on endothelial cells and vascular smooth muscle cells.

Caffeine has various pharmacological actions including: (1) action of an antagonist of adenosine receptors, (2) inhibition of phosphodiesterase (PDE), (3) increase in intracellular calcium concentration, (4) production of EDHF, (5) decrease in oxidative stress, and (6) enhancement of eNOS expression for the regulation of vascular function (Figure 1).

Several investigators have shown that caffeine is an antagonist of adenosine receptors [129,130]. Adenosine is well known to be a potent vasodilator and a modulator of cardiac function. These actions are mediated by four different G protein-coupled receptors, namely adenosine A_1_, A_2A,_ A_2B_, and A_3_ receptors [131]. The vasodilatory action of adenosine is mediated by induction of NO release from endothelial cells via the adenosine A_2A_ receptor [132,133]. Caffeine acts as an antagonist for the adenosine A_2A_ receptor, leading to a decrease in NO production that results in endothelial dysfunction. Indeed, Smits et al. reported that intra-brachial infusion of adenosine increased FBF, and that intra-brachial infusion of caffeine blocked the FBF response to adenosine [129]. Bruce et al. showed that acute oral administration of 200 mg of caffeine decreased exhaled levels of the biochemical marker NO [36]. On the other hand, adenosine-induced vasoconstriction is mediated by a decrease in NO release from endothelial cells via the adenosine A_1_ receptor [134]. Therefore, caffeine has the potential for increasing NO production through inhibition of the adenosine receptor A_1_, leading to augmentation of endothelial function. Umemura et al. showed that although oral administration of caffeine did not alter baseline FBF, ACh-induced vasodilation was significantly increased in the caffeine group. L-NMMA completely abolished caffeine-induced augmentation of endothelium-dependent vasodilation [88]. These findings suggest that oral administration of 300 mg of caffeine augments endogenous NO production by predominately inhibiting adenosine receptor A_1_ compared with inhibition of the adenosine receptor A_2A_. Different binding affinities of caffeine or different doses of caffeine to adenosine receptors A_1_ and A_2A_ may decide the direction, either diminishment or augmentation, or no change in endothelial function.

Increases in intracellular free calcium concentration induced by an increase in shear stress by agonists binding to G-protein-coupled receptors and by receptor-independent agonists activate the calcium/calmodulin-dependent protein kinase, leading to an increase in eNOS activity [135,136]. Hatano et al. showed that caffeine promotes NO synthesis in the endothelium through the release of calcium from the endoplasmic reticulum via activation of the ryanodine-sensitive calcium channel, resulting in caffeine-induced augmentation of endothelial function [137]. It is thought that caffeine increases the eNOS activity through an increase in the intracellular free calcium level by adenosine receptors-related increase in calcium influx and the ryanodine receptors-related release of calcium from store sites.

Caffeine is also a non-selective inhibitor of phosphodiesterases (PDEs), which are involved in the intracellular signaling pathway [138]. PDE5, one of the members of the family of PDEs, inhibits the degradation of cyclic guanosine monophosphate (cGMP), a second messenger of NO, to 5’GMP [139]. An increase in the level of intracellular cGMP is known to activate protein kinases A and G, leading to a decrease in intracellular free calcium concentrations, which results in vascular smooth muscle relaxation (vasodilation) [140]. It has been shown that caffeine-induced augmentation of endothelial function is due to the suppression of cGMP degradation in isolated rat aortas [137]. Caffeine inhibits PDEs, which leads to the accumulation of cAMP, resulting in an increase in non-contractile free calcium concentrations, decrease in intracellular free calcium concentrations, and inhibition of myosin light chain kinase phosphorylation in vascular smooth muscle cells [141]. In addition, caffeine directly inhibits myosin light chain kinase phosphorylation and the interaction of myosin and actin [142]. Both endogenous NO released from the endothelium and exogenous NO act on vascular smooth muscle cells. Therefore, to discuss endothelium-dependent vasodilation as a reflection of endothelial function, it is important to consider the condition of endothelium-independent vasodilation reflecting vascular smooth muscle function.

In general, EDHF is released from the endothelium via stimulation through shear stress and the binding of agonists to their receptors, and then EDHF opens calcium-activated potassium channels in vascular smooth muscle cells, resulting in relaxation of vascular smooth muscle via hyperpolarization of the cell membrane [143]. Caffeine is known to release calcium from the intracellular calcium store sites in endothelial cells and vascular smooth muscle cells through ryanodine receptors [137,144]. An increase in the intracellular free calcium concentration induced by caffeine results in the production of EDHF from the endothelium. EDHF, as well as NO, plays an important role in the regulation of endothelial function, especially in the microvasculature [145]. The effects of caffeine on endothelial function assessed using RH responses in studies may reflect the EDHF-related endothelial function in the microvasculature. Caffeine not only activates, but also inhibits, various types of calcium and potassium channels on the cell membrane in endothelial cells and vascular smooth muscle cells [144,146]. In any case, it is likely that the effects of caffeine on these channels are complex and differ depending on doses of caffeine and types of cells, including endothelial cells, vascular smooth muscle cells, cardiomyocytes, and neural cells of different species.

In many clinical studies, the acute and chronic effects of caffeine ingestion on oxidative stress markers, including 8-isoplostane and malondialdehyde-modified low density lipoprotein, have been investigated [5,147]. Most studies have shown that caffeine ingestion decreases levels of oxidative stress markers. There has been no study showing the harmful effects of caffeine on oxidative stress biomarkers. Caffeine prevents the production of ROS via its ability to scavenge ROS [148]. These findings suggest that an increase in NO bioavailability via the inhibition of ROS contributes to the improvement of endothelial function after caffeine consumption. The anti-oxidative system also plays an important role in the regulation of endothelial function through a relative increase in NO bioavailability [149]. It has been shown that caffeine improves the oxidative defensive system in animal models and in humans [150,151]. Acute and chronic caffeine ingestion increased the levels of anti-oxidant system markers including glutathione, superoxide dismutase, and catalase, and the total antioxidant capacity [5,147,152,153]. However, some studies showed that caffeine had no effect on the anti-oxidant system or that caffeine decreased levels of anti-oxidant system biomarkers [153,154]. Although the effects of caffeine on the anti-oxidant system are controversial, protective effects of caffeine against the oxidative stress in animal models and in humans have been confirmed.

It has been hypothesized that caffeine is a vasoconstrictive substance [9,10,11,18,19,20,21]. Administration of 300 mg of caffeine elevated blood pressure in healthy subjects, and administration of 200 mg of caffeine elevated blood pressure in patients with CAD but not in subjects without CAD, suggesting that caffeine has vasoconstrictive effects [11]. These results support results of previous studies showing that acute administration of caffeine elevates peripheral blood pressure [18,19,20,21]. Karatzig et al. reported that central blood pressure was increased after acute administration of caffeine, while peripheral blood pressure did not significantly change [155]. Various factors including hypertension, exercise, and age affect the blood pressure response to caffeine [156]. These observations suggest that confounding factors should be kept reasonably constant for the assessment of changes in blood pressure during caffeine administration. Caffeine acts as an antagonist for adenosine A_2A_ receptors, resulting in the elevation of peripheral vascular resistance [129,130]. The caffeine-induced pressor effect is thought to be due to the elevation of peripheral vascular resistance rather than an increase in cardiac output [18]. The elevation of peripheral vascular resistance induced by caffeine may involve several mechanisms, of which the most plausible is antagonism of adenosine, leading to vasoconstriction and increased total peripheral resistance [19,21]. It is not clear how a balance of caffeine-induced augmentation of endothelial function and caffeine-induced elevation of blood pressure affects the progression of atherosclerosis and CV morbidity and mortality.

There is an interaction between sympathetic nerve activity and endothelial function [157]. It is well known that the mechanisms by which caffeine increases sympathetic nerve activity are a direct effect and an increase in the release of catecholamines through inhibition of the adenosine receptor [158,159,160]. In addition, it has been shown that in healthy young subjects, a single dose of caffeine of 250 mg increases plasma renin activity, leading to activation of sympathetic nerve activity [161]. Some previous studies showed that caffeine consumption decreases heart rate or does not alter heart rate, while caffeine elevates blood pressure [20,162,163]. Caffeine acutely increases sympathetic nerve activity [20] but it seems reasonable to assume that the elevation of blood pressure induces a baroreceptor-mediated inhibition of cardiac sympathetic activity, resulting in suppression of the increase in heart rate. It remains unclear whether the caffeine-induced increase in sympathetic nerve activity affects vascular function assessed using physiological function tests.

### 5.3. Tea

All of the studies using black tea or green tea showed significant augmentation of FMD in healthy subjects and significant improvement of FMD in patients with CVD [80,81,82,83,84,85,86]. Tea has many flavonoids with anti-oxidant properties, including polyphenols, catechins, and isoflavones [1,2]. Even under the condition of the presence of harmful effects of other compounds (including caffeine) on endothelial function, flavonoids and beneficial effects of the anti-oxidant ability of caffeine may surpass the harmful effects of other compounds on endothelial function. However, the possibility of publication bias due to the difficulty in submitting negative results cannot be ruled out.

## 6. Conclusions

It is expected that the ingestion of coffee and caffeine has beneficial effects on endothelial function through enhancement of NO bioavailability. However, the effects of coffee and caffeine on endothelial function and CV events remain controversial. Unfortunately, the number of studies in which the effects of coffee or caffeine on vascular function were evaluated has been small. Although some studies were conducted as double-blind, randomized, placebo, cross-over, and active drug studies, the number of subjects was also small. Most of the subjects in studies were healthy subjects and few studies were conducted in subjects with high CV risk factors and patients with CVD. Further studies are needed to assess the long-term effects of coffee or caffeine on vascular function and CV events in a large population, including subjects with high CV risk factors and patients with a history of myocardial infarction or stroke.

## Figures and Tables

**Table 1:** Effects of coffee and caffeine on endothelial function.

Study (ref.)	Coffee (Contents)/Caffeine	Participants (Number)	Follow-Up Period	Endothelial Function Test	Results
[45]	Coffee (caffeine 100 mg)/Caffeine 200 mg	Healthy subjects (*n* = 12; 4 men, 8 women)	1 h	Exhaled NO levels	Both coffee and caffeine deceased exhaled NO levels.
[80]	Black tea (caffeine 200 mg)	CAD patients (*n* = 66)	2 h, 4 weeks	FMD	Black tea improved FMD in brachial artery, while an equivalent dose of caffeine did not alter FMD. Both black tea and caffeine did not alter NID.
[81]	Black tea (caffeine ≈250 mg)	Hyperlipidemia patients (*n* = 21)	4 weeks	FMD	Black tea improved FMD and NID in brachial artery.
[82]	Black tea (caffeine ≈50 mg)	CAD patients (*n* = 20)	4 h	FMD	Black tea improved FMD and NID in brachial artery after meal loading, while black tea alone did not alter both FMD and NID.
[46]	Coffee (caffeine 80 mg)	Healthy young adults (*n* = 17; 9 men, 11 women)	2 h	FMD	Caffeinated coffee decreased FMD in brachial artery, while decaffeinated coffee did not alter FMD.
[77]	Caffeine 300 mg	Healthy young men (*n* = 20)	1 h	ACh-induced vasodilation	Caffeine augmented ACh-induced vasodilation in forearm tissue and did not alter SNP-induced vasodilation.
[31]	Coffee (caffeine 83–373 mg)	Healthy women (*n* = 730), diabetic women (*n* = 663)	Cross-sectional	Chemical biomarkers	Coffee consumption is inversely correlated with markers of endothelial dysfunction.
[83]	Black tea (caffeine 100 mg)	Healthy men (*n* = 19)	1 week	FMD	Black tea dose-dependently augmented FMD in brachial artery.
[32]	Coffee (CGA 140 mg)	Healthy men (*n* = 20)	3 months, 4 months	RH ratio	Coffee polyphenols improved RH ratio in forearm tissue after glucose loading.
[33]	Coffee (CGA 134 mg–300 mg, caffeine 59–70 mg, HHQ 0.03–0.12 mg)	Subjects with CV risk factors (*n* = 21)	8 weeks	FMD	Coffee improved FMD in brachial artery.
[79]	Green tea (caffeine 125 mg)/Caffeine 125 mg	Healthy subjects (*n* = 14)	2 h	FMD	Green tea augmented FMD in brachial artery, while an equivalent dose of caffeine did not alter FMD.
[84]	Black tea (caffeine 125 mg)	Healthy women (*n* = 16)	2 h	FMD	Black tea augmented FMD in brachial artery and did not alter NID.
[34]	Coffee (2 cups of decaffeinated)	Healthy subjects (*n* = 15; 8 men, 7 women)	1 h	FMD	Decaffeinated coffee increased FMD in brachial artery.
[47]	Coffee (Italian espresso 25 mL)	Healthy subjects (*n* = 20; 10 men, 10 women)	5–7 days	FMD	Caffeinated coffee decreased FMD in brachial artery, while decaffeinated coffee did not alter FMD.
[78]	Caffeine 200 mg	Subjects without CVD (*n* = 40) and with CVD (*n* = 40)	1 h	FMD	Caffeine increased FMD in brachial artery and did not alter NID.
[35]	Coffee (boiled Greek coffee, caffeine 56–126 mg)	Elderly subjects (*n* = 142)	Cross-sectional	FMD	Greek type of coffee had higher increased FMD in brachial artery compared to other groups.
[36]	Coffee (CGA)	Healthy men (*n* = 15)	1.5 h	RH index	Coffee polyphenols improved RH index in finger tips after glucose loading.
[37]	Coffee (CGA)	Healthy men (*n* = 13)	2 h	FMD	Coffee polyphenols improved FMD in brachial artery after meal loading.
[38]	Coffee (CGA 355 mg, caffeine 54.7 mg)	Healthy men (*n* = 19)	3 h	FMD	Coffee polyphenols improved FMD in brachial artery after meal loading.
[39]	Coffee (caffeine 54.5 mg)	Healthy subjects (*n* = 27; 13 men, 14 women)	75 min	RH flow	Caffeinated coffee augmented reactive hyperemia of finger blood flow, while decaffeinated coffee did not alter reactive hyperemia of finger blood flow.
[48]	Coffee (caffeine 240 mg)	Healthy subjects (*n* = 19; 11 men, 8 women)	4 h	RH index	Coffee did not alter RH index in finger tips.
[49]	Coffee (CGA 420–780 mg, caffeine 193 mg)	Healthy subjects (*n* = 74; 37 men, 37 women)	1 h, 8 weeks	FMD	Coffees containing CGA did not alter FMD in brachial artery.
[50]	Coffee (CGA 450–900 mg)	Healthy subjects (*n* = 16; 6 men, 10 women)	1 h, 4 h	FMD	Coffees containing CGA did not alter FMD in brachial artery.
[40]	Coffee (CGA 89–310 mg, caffeine 110 mg)	Healthy men (*n* = 15)	5 h	FMD	Coffees containing CGA increased FMD in brachial artery.
[85]	Black tea (caffeine 37.3 mg)	Hypertension (*n* = 19; 7 men, 12 women)	8 days	FMD	Black tea improved FMD in brachial artery and increased circulating progenitor cells.
[41]	Coffee (CGA 300 mg)	Healthy men (*n* = 12)	2 h	FMD	Caffeinated coffee containing CGA increased FMD in brachial artery, while decaffeinated coffee containing CGA did not alter FMD.
[42]	Coffee (caffeine 78 mg)	Healthy subjects (*n* = 16; 8 men, 8 women)	1.5 h	ACh-induced vasodilation	Caffeinated coffee augmented ACh-induced vasodilation in the forearm skin, while decaffeinated coffee did not alter ACh-induced vasodilation.
[43]	Coffee (CGA-enriched green coffee bean)	Healthy men (*n* = 16)	2 weeks	FMD	CGA-enriched green coffee bean increased FMD in brachial artery.
[86]	Black tea (3 Lipton tea bags)	Healthy young adults (*n* = 17, 7 men, 10 women)	4 weeks	FMD	Black tea augmented FMD in brachial artery.
[44]	Coffee (CGA 373–412 mg, caffeine 59–75 mg, HHQ 0.10–0.76 mg)	Stage 1 hypertension (*n* = 37; 26 men, 11 women)	1 week	FMD	Caffeinated coffee containing high content of CGA and low content of HHQ improved FMD in brachial artery after meal loading.

CAD indicates coronary artery disease; NO, nitric oxide; FMD, flow-mediated vasodilation; NID, nitroglycerine-induced vasodilation; ACh, acetylcholine; SNP, sodium nitroprusside; CGA, chlorogenic acids; HHQ, hydroxyhydroquinone; CV, cardiovascular; CVD, cardiovascular disease; RH, reactive hyperemia.
